# Attenuated NoGo-related beta desynchronisation and synchronisation in Parkinson’s disease revealed by magnetoencephalographic recording

**DOI:** 10.1038/s41598-019-43762-x

**Published:** 2019-05-10

**Authors:** Hung-Ming Wu, Fu-Jung Hsiao, Rou-Shayn Chen, Din-E Shan, Wan-Yu Hsu, Ming-Chang Chiang, Yung-Yang Lin

**Affiliations:** 1 Institute of Brain Science, National Yang-Ming University, Taipei, Taiwan; 2 Brain Research Center, National Yang-Ming University, Taipei, Taiwan; 3 School of Medicine, National Yang-Ming University, Taipei, Taiwan; 4 Department of Neurology, Neurological Institute, Taipei Veterans General Hospital, Taipei, Taiwan; 5 Department of Neurology, Taipei Hospital, Ministry of Health and Welfare, New Taipei City, Taiwan; 6 Department of Neurology, LinKou Chang Gung Memorial Hospital, Taoyuan, Taiwan; 7 Department of Neurology, University of California San Francisco, San Francisco, California United States of America; 8 Neuroscape, University of California San Francisco, San Francisco, California United States of America; 9 Department of Biomedical Engineering, National Yang-Ming University, Taipei, Taiwan

**Keywords:** Cognitive control, Parkinson's disease

## Abstract

Parkinson’s disease (PD) is a neurodegenerative disorder characterised by motor abnormalities. Many non-demented patients with PD have cognitive impairment especially in executive functions. Using magnetoencephalographic (MEG) recording combined with event-related desynchronisation/synchronisation (ERD/ERS) analysis, we investigated cortical executive functions during a Go/NoGo task in PD patients and matched healthy subjects. PD patients had a longer reaction time in the Go condition and had a higher error ratio in both Go and NoGo conditions. The MEG analysis showed that the PD patients had a significant reduction in beta ERD during the NoGo condition and in beta ERS during both Go and NoGo conditions compared with the healthy subjects (all p < 0.05). Moreover, in the Go condition, the onsets of beta ERD and ERS were delayed in PD patients. Notably, NoGo ERS was negatively correlated with the Unified Parkinson’s Disease Rating Scale (UPDRS) score in PD patients. The present study demonstrated abnormalities in motor programming, response inhibition, and frontal inhibitory modulation in PD. Further extensive investigations are necessary to confirm the longitudinal treatment responses in PD.

## Introduction

Parkinson’s disease (PD) is a neurodegenerative disease with predominant motor dysfunction, which is characterised by bradykinesia, rigidity, tremor, and postural instability. PD patients could also have cognitive problems, even with normal Mini-Mental State Examination (MMSE) scores^1^. These non-motor symptoms, including deficits in executive functions, language, memory, and visuospatial skills, have been reported in the early stages of PD^2^. Executive functions are necessary for the cognitive control of behaviour, including attentional control, inhibitory control, working memory, and cognitive flexibility, as well as reasoning, problem solving, and planning^3^. Impairments in executive functions have been considered a dominant signature reflecting functional abnormalities in fronto-striatal circuits^4,5^. Accumulated neuropsychological evidence^3,6^ has demonstrated impaired executive functions in PD patients with the use of the Wisconsin Card Sorting Test, delayed response tasks, Stroop test, and Go/NoGo task^2^. Previous pharmacological^7,8^ and electrophysiological^9^ studies have demonstrated altered executive functions in PD. Moreover, impairment in executive functions was shown to be associated with the development of dementia in PD patients^10^.

The Go/NoGo task has been used to measure sustained attention and response inhibition, which are essential components of executive functions^3,11^. During the task, subjects are asked to make a motor response (Go) or withhold the response (NoGo) according to visual or auditory cues. Compared with healthy controls, PD patients have a slower response in the reaction time task due to decreased capability in cognitive responses^12^. Moreover, their performance is poorer in cued reaction time tasks than in uncued reaction time tasks, which could be attributed to PD patients failing to use the cue to prepare a response^13^. If a warning cue is presented sufficiently prior to the Go stimuli, the proactive inhibitory control (prevent prepotent responses to potentially inappropriate stimuli) has been released at Go stimuli occurrence, and automatic responses are generated^14^. However, PD patients would have impairment in dynamic switching from proactive inhibitory control to sensorimotor processing^15^. Event-related potentials (ERP) recording further showed decreased amplitude and delayed latency in the NoGo-related frontocentral N2 and P3 components, indicating a deficit in response inhibition in PD^16^. Moreover, with regard to the frequency-specific characteristics of high-order processes for executive and inhibitory functions, more information can be expected when cognitive and motor processes in PD during a Go/NoGo task are examined using the event-related desynchronisation (ERD) and synchronisation (ERS) methods, such as that the ERD and ERS are more directly related to movement programming than ERP^17,18^.

Movement-related ERD/ERS, which explicitly characterise cortical motor preparation, execution, and inhibition^19^, were attenuated in PD, and the delayed onset of ERD and the reduction in the amplitude of ERS were substantially correlated with the severity of motor symptoms^20–22^. This indicates a relationship between disruption in dopaminergic neurotransmission in the basal ganglia and impairment in motor preparation and programming. Moreover, task-related ERD/ERS, implicitly encoding information and cognitive processes, reflected oscillatory alterations in response to working memory^23^ and auditory discrimination^24^ tasks for cognitive deficits in PD. Notably, beta oscillations were associated with response preparation and inhibition^17,25,26^, cognitive function, and attentional control^26,27^. Recording of the local field potentials from the subthalamic nuclei (STN) revealed that beta ERD started prior to (both Go and NoGo) stimuli and continued for several hundred milliseconds, and then an ERS occurred in both Go and NoGo conditions, but the NoGo ERD was prematurely terminated compared to Go ERD and followed an earlier NoGo ERS^17^. The Go and NoGo ERD could be related to the cognitive process (response preparation) shortly before Go/NoGo stimulus; however, after the Go/NoGo stimulus, the Go ERD/ERS might be involved in movement processes, and NoGo ERD/ERS might be involved in inhibition processes^18^. Go ERD occurs over the contralateral frontal-medial and sensorimotor regions before a movement (a marker of movement preparation) and is followed by a large peri-movement ERD over bilateral sensorimotor areas^28^. When the movement ends, the beta ERD is followed by a post-movement beta ERS over the contralateral sensorimotor area^19,28^. In the NoGo condition, ERD occurs over bilateral sensorimotor regions and starts at the same time as the Go condition and contralateral beta ERS also occurs when prepared movements are terminated^28,29^. Attenuated Go ERD/ERS have been reported in patients with PD^22^. Because no muscle contractions are elicited in the NoGo condition, the corresponding ERD/ERS power is better for evaluating the cognitive processes than in the Go condition. However, the NoGo ERD/ERS activities in PD patients require further investigation.

As for motor and cognitive problems in PD, the Go/NoGo task combined with ERD/ERS analysis would elucidate cortical oscillatory alterations representative of underlying abnormal bottom-up/top-down motor function, inhibitory controls, and cognitive processes. Moreover, magnetoencephalographic (MEG) recordings, with their excellent temporal and spatial resolution, provide the neural correlates of spatio-temporal oscillatory patterns^30,31^. Our aim was to evaluate the difference in channel-based ERD/ERS activities in the sensorimotor region and its cortical involvement between PD patients and healthy controls. We hypothesise that in the Go and NoGo conditions, patients with PD are characterised by attenuated and delayed beta ERD and ERS, as well as poor behavioural performance with respect to reaction time and accuracy. Moreover, cortical oscillatory activities could correlate with clinical severity in PD.

## Results

### Demography and behavioural responses to perform the Go/NoGo task

The demographic and clinical data of PD patients are listed in Table 1. No significant difference was found between the PD patients and Healthy controls (HC) with respect to age or gender (both p > 0.1). The MMSE score was slightly lower in PD patients than in HC but did not reach a significant level (PD: 28.08 ± 1.93; HC: 29.15 ± 1.07; p = 0.225).

**Table 1:** Demographic data of patients with Parkinson’s disease.

No.	Age	Gender	Disease duration (years)	L-dopa dose (/day)	Stage	UPDRS	MMSE
1	54	M	20	1000 mg	II-III	31	26
2	66	M	7	600 mg	II	15	28
3	58	M	1	300 mg	II	20	30
4	66	M	8	600 mg	II	23	26
5	74	F	4	300 mg	I	19	30
6	66	M	7	300 mg	I	16	26
7	63	M	10	600 mg	II-III	36	30
8	74	F	4	300 mg	I	20	26
9	64	M	5	300 mg	II	21	29
10	74	M	10	300 mg	II	25	30
11	77	M	5	300 mg	II	18	30
12	53	F	6	300 mg	II	29	26

Note: UPDRS: Unified Parkinson’s Disease Rating Scale. MMSE: Mini-Mental State Examination.

In the Go condition, the PD patients had a longer reaction time (PD: 742.86 ± 158.45 ms; HC: 532.03 ± 61.43 ms; p < 0.001) and a higher error rate (PD: 28.50 ± 22.05%; HC: 4.51 ± 4.95%; p = 0.001) compared to HC. In the NoGo condition, the PD patients also had a higher error rate (PD: 17.39 ± 21.94%; HC: 2.51 ± 2.60%; p = 0.022).

### Beta ERD and ERS responses during the Go/NoGo task

**Figure 1:**
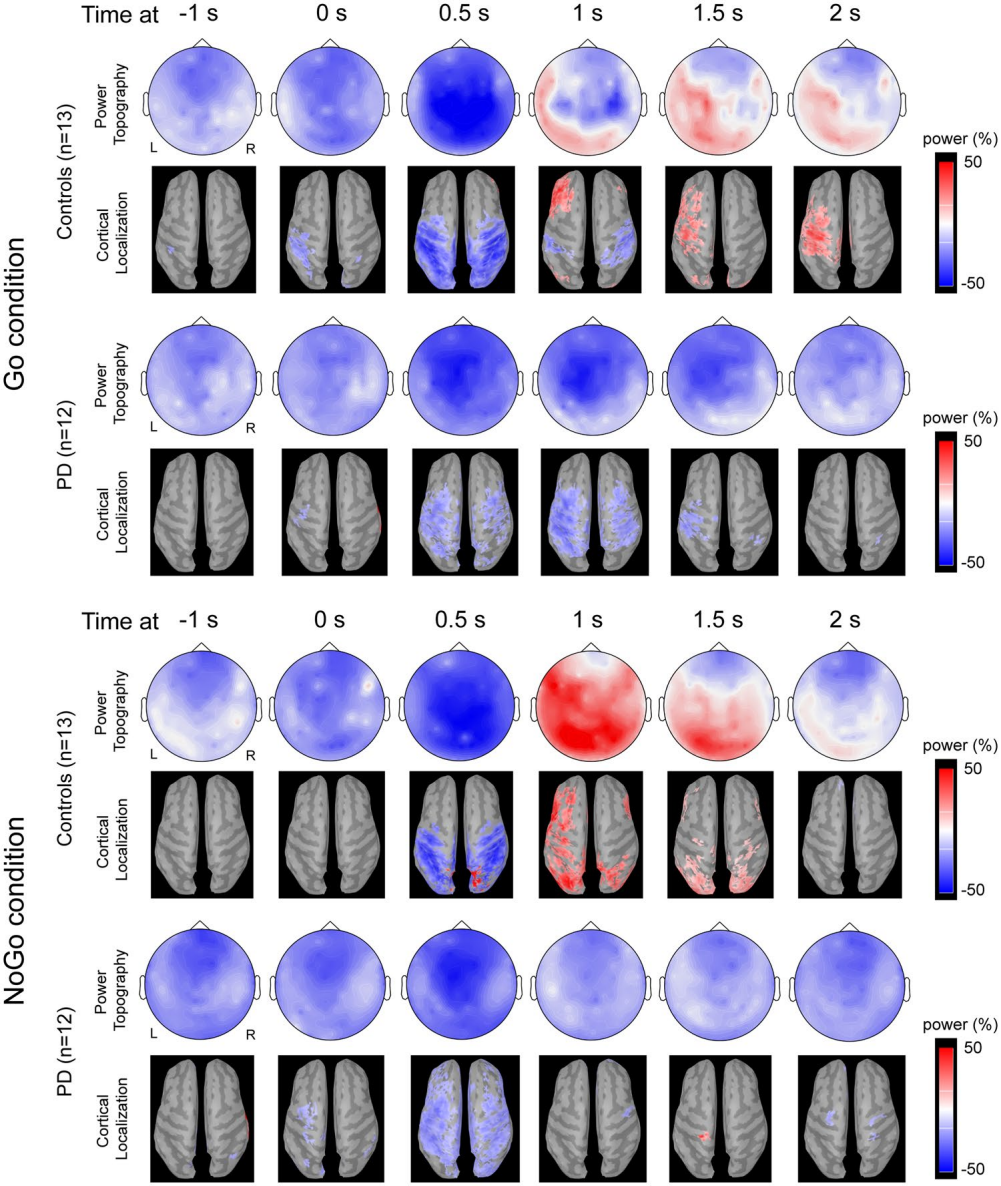
The grand-average time-varying topographic distribution of peak beta oscillatory activity in the Go and NoGo conditions are shown for healthy controls and PD patients. Power change is colour-coded; increase is denoted with red and decrease with blue.

The grand-average time-varying topographic distributions and cortical localisations of oscillatory activities at the beta band for healthy controls and PD patients are illustrated in Fig. 1. For the Go condition, the beta ERD in healthy controls started over left fronto-medial and sensorimotor regions in the range from −1 s to 0 s and was localised in the left post-central gyrus. The ERD then extended to bilateral frontal and sensorimotor and parietal regions and was localised in the bilateral pre-central and post-central gyri. The ERS was prominent in the left sensorimotor and parietal regions and localised in the left pre-central and post-central gyri in the range from 1.5 s to 2 s. Compared to the healthy controls, the PD patients had a similar topography response and source localisation before and peri-movement but lack ERS. For the NoGo condition, the healthy controls had prominent ERD over bilateral frontal and sensorimotor and parietal regions at 0.5 s, localised in the bilateral pre-central, post-central gyri, posterior parietal areas, and occipital lobes. The ERS was prominent over the left lateral frontal and frontal regions and bilateral sensorimotor and parietal regions approximately 1 s and localised in the left dorsolateral prefrontal cortex and premotor cortex and pre-central and post-central gyrus and bilateral posterior parietal areas and bilateral occipital lobes. In PD patients, the ERD topography response was similar to healthy controls, but the source localisation was more widely distributed and extended to the bilateral premotor areas compared to healthy controls. In addition, the PD patients did not have prominent ERS in topography response, and the source was localised only over the left post-central gyrus at 1.5 s.

**Figure 2:**
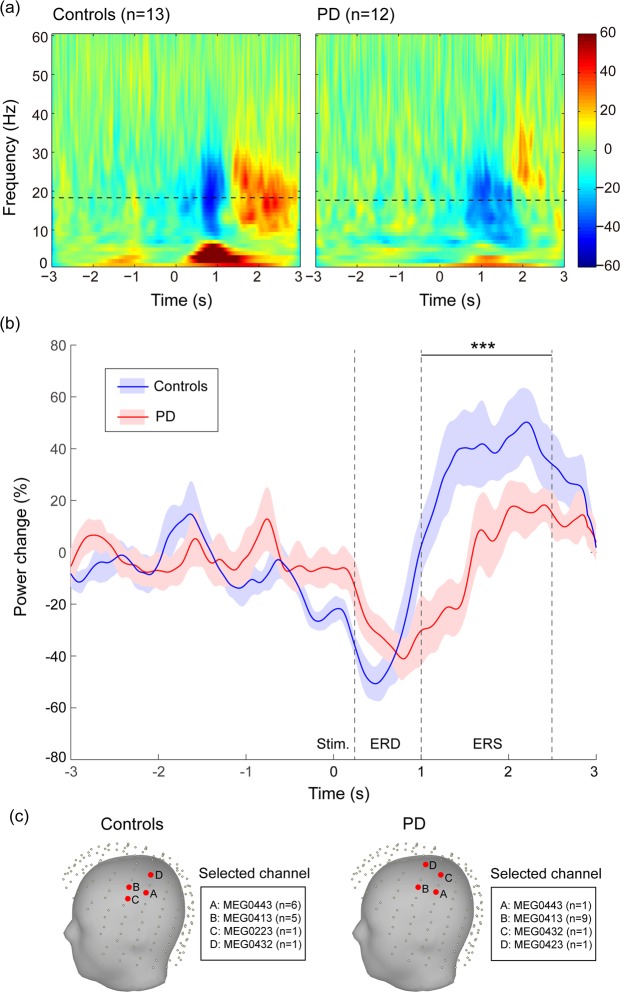
In the Go condition, (**a**) grand-average time-frequency representations of oscillatory activities are exhibited for healthy controls and PD patients, (**b**) dynamics of grand-average peak-beta power across subjects with the standard error shown for controls and PD patients. (**c**) Representative channels in all subjects. Stim., onset of visual stimulus; ERD, event-related desynchronisation; ERS, event-related synchronisation; ***p < 0.001.

In the Go condition, the grand-average time-frequency plots of oscillatory activities in the representative channel where the largest task-related beta oscillation was observed are illustrated for HC and PD patients in Fig. 2(a). Clear beta ERD and ERS were observed after the Go stimuli (at time 0 s) and time-varying oscillatory power change with its standard error at peak beta frequency is shown in Fig. 2(b). The mean power changes at the time interval of 0.25–1 s and 1–2.5 s were extracted for ERD and ERS responses, respectively. The amplitude of ERS was significantly smaller in PD patients than HC (PD: −4.11 ± 24.67; HC: 37.42 ± 33.82; p = 0.002), whereas no significant difference in the amplitude of ERD responses was detected (PD: −33.27 ± 24.19; HC: −38.97 ± 29.67; p = 0.406). The peak frequency of beta activities was not significantly different between PD patients and HC (PD: 17.41 ± 3.23 Hz, HC: 18.38 ± 3.43 Hz; p = 0.476).

**Figure 3:**
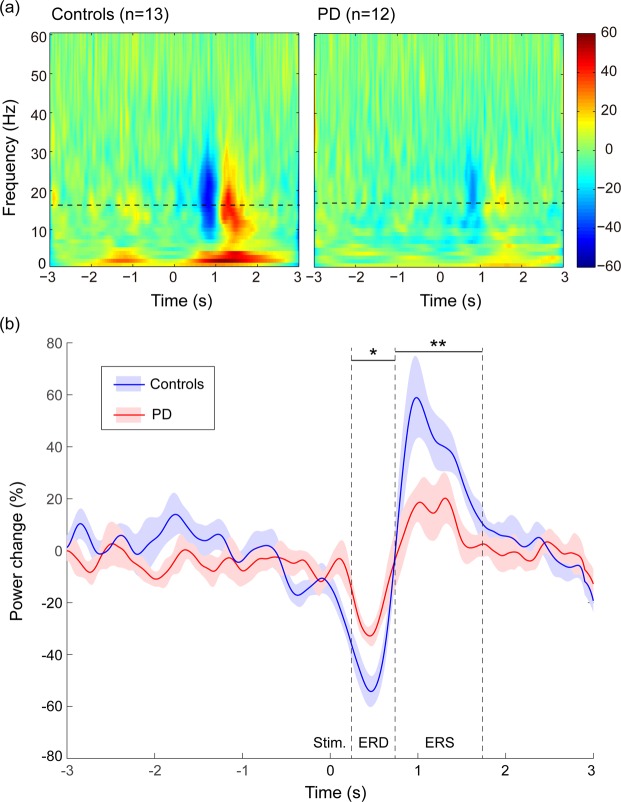
In the NoGo condition, (**a**) grand-average time-frequency plots of oscillatory activities are shown for healthy controls and PD patients, (**b**) dynamics of grand-average peak-beta power across subjects with the standard error are shown for controls and PD patients. Stim., onset of visual stimulus; ERD, event-related desynchronisation; ERS, event-related synchronisation; *p < 0.05; **p < 0.01.

For the NoGo condition, the grand-average time-frequency plots of oscillatory activities are shown in Fig. 3(a). Clear beta ERD and ERS were observed after the visual cue (at time 0 s), and time-varying oscillatory power change with its standard error at peak beta frequency is shown in Fig. 3(b). The mean power changes at the interval of 0.25–0.75 s and 0.75–1.75 s were calculated as the intensity of ERD and ERS responses, respectively. Both the amplitude of ERD (PD: −24.29 ± 13.42; HC: −42.27 ± 21.06; p = 0.017) and ERS (PD: 10.11 ± 17.66; HC: 36.12 ± 26.29; p = 0.008) were significantly smaller in PD patients than in HC. No significant difference in peak beta frequency was observed between the patients and controls (PD: 18.50 ± 2.81 Hz, HC: 16.61 ± 2.14 Hz; p = 0.123).

There was a significant difference between the PD patients and HC in the onset latencies of the ERD and ERS responses during the Go condition (ERD, p = 0.043; ERS, p = 0.003) (Table 2). This indicates that task-related oscillatory activities were delayed in PD. However, in the NoGo condition, the onset latencies of ERD and ERS were not significantly different between the patients and controls (ERD, p = 0.119; ERS, p = 0.188). According to our criteria of onset latency measurement (please refer to the Materials and Methods section; the onset latency of ERD and ERS is respectively defined as the first of 5 consecutive, significant values less and larger than the mean power during the reference period), the onset latency of NoGo ERS in Cases no. 8 and 10 was undetectable.

**Table 2:** Onset latencies of ERD and ERS in the Go/NoGo task.

	Onset latency of ERD (s)		Onset latency of ERS (s)	
	HC	PD	HC	PD
Go	−0.71 ± 0.38	−0.33 ± 0.46	1.23 ± 0.38	1.74 ± 0.39
NoGo	−0.59 ± 0.44	−0.30 ± 0.41	0.79 ± 0.16	0.90 ± 0.24

The mean ± standard deviation is shown above. Negative numbers are defined as the onset latency before Go/NoGo stimuli. Note: ERD: event-related desynchronisation. ERS: event-related synchronisation. HC: healthy controls. PD: Parkinson’s disease.

### Correlations among clinical scores, behavioural data, and oscillatory activities

**Figure 4:**
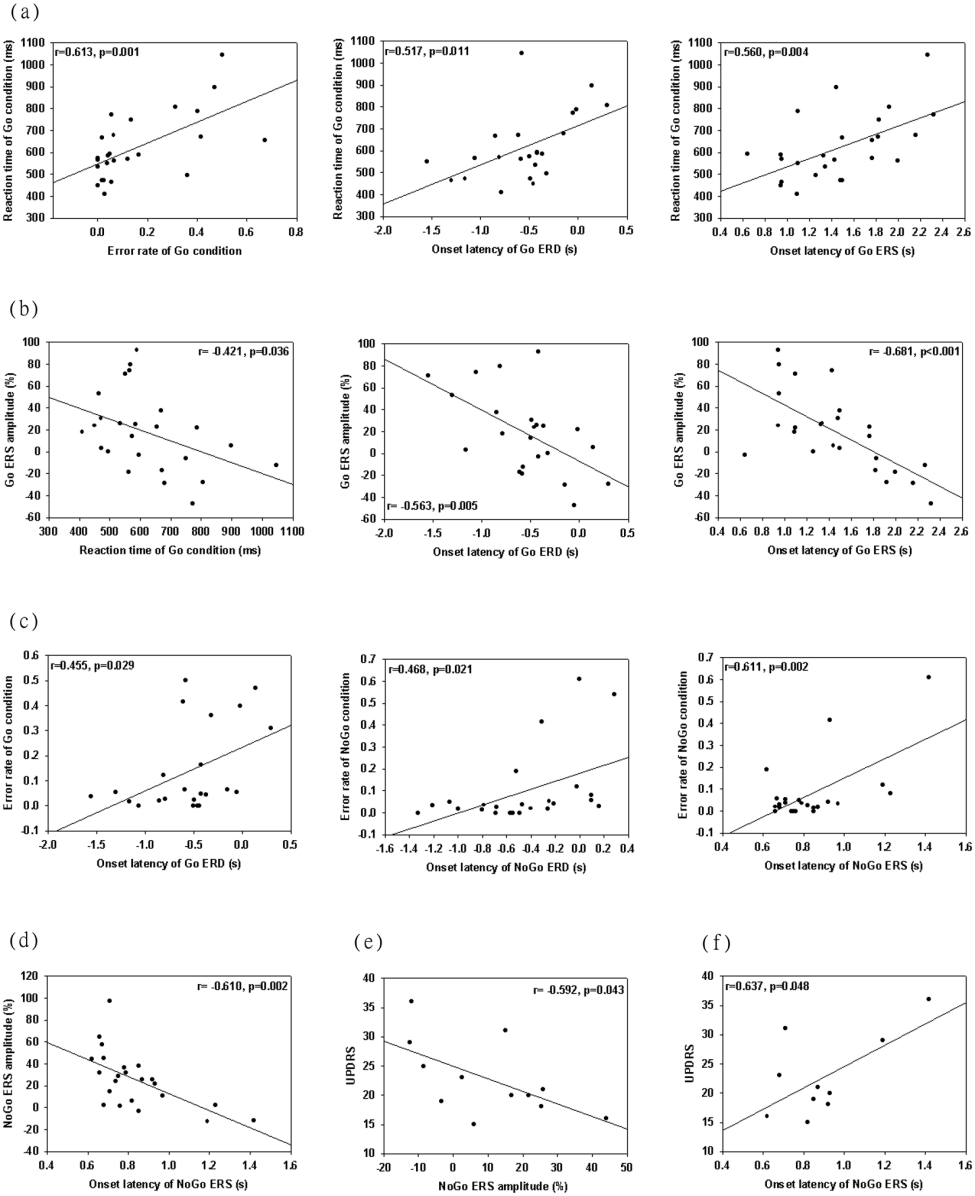
Correlations among clinical scores, behavioural data, and oscillatory activities. For the patients and controls, (**a**) reaction time is positively correlated with the error rate in the Go condition and the onset latency of Go ERD and Go ERS. (**b**) The Go ERS power is negatively correlated with the reaction time and the onset latency of Go ERD and Go ERS. (**c**) The error rate in the Go condition is positively correlated with the onset latency of Go ERD, and the NoGo error rate is positively correlated with the onset latency of NoGo and NoGo ERS. (**e**) The power of NoGo ERS is negatively correlated with the onset latency of NoGo ERS. For the patients, (**f**) UPDRS score is negatively correlated with NoGo ERS power and is positively correlated with the onset latency of NoGo ERS. ERD, event-related desynchronisation; ERS, event-related synchronisation; UPDRS, Unified Parkinson’s Disease Rating Scale.

For the patients and controls, in the Go condition, the reaction time was positively correlated with the error rate (r = 0.613, p = 0.001) and the onset latency of ERD (r = 0.517, p = 0.011) and ERS (r = 0.56, p = 0.004) (Fig. 4). The ERS power was negatively correlated with the reaction time (r = −0.421, p = 0.036) and the onset latency of ERD (r = −0.563, p = 0.005) and ERS (r = −0.681, p < 0.001). Moreover, there was a significant correlation between the onset latency of ERD and the error rate (r = 0.455, p = 0.029). In the NoGo condition, the error rate was positively correlated with the onset latency of ERD (r = 0.468, p = 0.021) and ERS (r = 0.611, p = 0.002). The onset latency of ERS was also correlated with the power of ERS (r = −0.61, p = 0.002).

For the PD patients, there was a significant correlation between the UPDRS score and the amplitude (r = −0.592, p = 0.043) and onset latency (r = 0.637, p = 0.048) of ERS in the NoGo condition. There was no significant correlation of oscillatory activities with the MMSE score, disease duration, or dose of the medication (all with a p-value > 0.05).

## Discussion

In this study, altered executive function, manifested both behaviourally and neurophysiologically, was noted in PD patients. The PD patients had a longer reaction time in the Go condition and had a higher error rate in both the Go and NoGo conditions. The power responses of beta ERD in the NoGo condition and beta ERS in both the Go and NoGo conditions in the PD patients were also reduced. Furthermore, in the Go condition, the onset latencies of ERD and ERS responses were both delayed. Notably, NoGo elicited ERS responses with respect to power and onset latency were associated with the UPDRS score in PD patients.

Performing choice response time experiments such as the Go/NoGo task depends upon not only sensorimotor processes but also cognitive control^12^. In line with previous findings^12,13,32^, patients with PD exhibited prolongation in response time and reduction in response accuracy, which could be ascribed mainly to cognitive deficits in attentional control and stimulus evaluation^12,13,32^. Moreover, the basal ganglia could be responsible for mediating the reaction time of a response task^32^ during the preparation, selection, and execution of learned motor work^33^. Consequently, disrupted interactions between the basal ganglia and Supplementary Motor Area could lead to dysfunction in motor execution^32^. In the current patient cohort, the alterations in the Go task could be related to the abnormal proactive inhibitory control and motor function, whereas the relatively poor performance in the NoGo task might imply the dysfunction of proactive inhibitory control and response inhibition.

In the Go condition, prominent contralateral beta ERD activities during movement preparation and execution were consistent with the previous literature^34,35^ and were related to movement preparation and cognitive selection of a proper motor response^36,37^. In the present study, patients with PD had a comparable amplitude but delayed onset of ERD responses relative to healthy controls, which was in accordance with the notion of a decrease in thalamo-cortical influx^38^ and consequent prolonged activity over the cortical projection zones in PD^21,39,40^. In line with this observation, it has been found that during task-related movements, the ERP activities at 200–600 ms^16,41^ and movement-related beta ERD responses^20^ in the PD patients were unaffected on the amplitude/power measures, but the latency responses were delayed, which was ascribed to inadequate motor planning^42^. Deep brain stimulation of the STN improved motor symptoms and decreased reaction time in Go condition in patients with PD^43^. Reaction time task is thought to maintain a proactive inhibition for preventing inappropriate response^44^, and correspondingly, prolonged the reaction time in PD patients could reflect a deficit in voluntary release of proactive inhibition^45^. Delayed onset of Go ERD could indicate PD patients need to take a longer time to achieve the sufficient cognitive process and then release the proactive inhibition. However, in PD, inconsistent findings of beta ERD power responses have also been reported and might stem from the compensation, improvement, or diminishment of motor function^22,46^. The task difficulty, such as the degree of directional uncertainty of the movement, correlated with the power of the beta ERD^47^, which might account for the discrepancy among studies. Taken together, the results showed that the onset latency in Go-related ERD responses could characterise the deficits in motor programming in PD because of the abnormal subcortical-cortical information transmission and cognitive processing of response selection.

In the NoGo condition, beta ERD activities were mainly observed over the sensorimotor cortex and were significantly attenuated in PD. The time interval of ERD responses (0.25–0.75 s) was assumed to reflect premotor and motor inhibition^48–50^, response conflict^51^, and evaluation of inhibitory processes^11,48,52–54^. Deterioration in response inhibition in PD was evidenced by attenuation in the amplitude of NoGo-related N2 or P3 activities^16,41,55^. The present findings are consistent with impaired inhibitory executive function in PD, which is associated with abnormal modulation in basal ganglia neurons^56^ due to the loss of dopamine^57,58^. The cognitive control network, connected the STN and inferior frontal cortex (IFC) and preSMA through cortico–subthalamic–pallidal–thalamo–cortical pathway^44^, were engaged in conflict situations^59^ and response inhibition^60^. The attenuated NoGo ERD in the motor cortex could reflect the malfunction of the active inhibitory process. Altogether, the aberrant patterns of ERD responses in PD might imply abnormalities in motor planning (delayed Go ERD) and response inhibition (attenuated NoGo ERD) under disrupted basal ganglia-cortical interactions.

Go ERS responses were related to the mechanism of cortical idling^61,62^, deactivation (activities returning to the baseline), or active inhibition^63^. The reduced and delayed beta ERS activities in PD are in agreement with EEG and MEG findings in the literature^20,22,64^ indicating loss of cortical inhibition after movement execution and therefore are associated with akinesia and poor motor control in PD. Stronger beta rebound has been observed as successful inhibition rather than unsuccessful inhibition^65^. Furthermore, after dopamine application, the motor functions, especially cortical inhibition in PD, were partially restored^66^. In combination with the evidence of attenuated NoGo ERD, the defective executive functions over the sensorimotor cortex in PD impede the inhibition of motor responses and cortical excitability.

In the NoGo condition, beta ERS following ERD was noted, which is consistent with previous findings in NoGo^29,65,67^ and motor imagery tasks^67,68^ and suggested that the phenomenon of neural synchronisation/desynchronisation could be elicited without movement execution and afferent sensory input. Furthermore, consistent with previous findings^29^, beta ERS in the NoGo condition occurred earlier compared to that in the Go condition (after movement execution) in both PD patients and HC. To our knowledge, this study first investigated the alterations of NoGo ERS activities in PD. Attenuated NoGo beta ERS responses were observed in our PD patients. Moreover, the onset latency of ERS was associated with the error rate of the behavioural performance and the power of the preceding ERD, indicating that NoGo ERS activities might reflect cognitive capabilities for attentional control and stimulus evaluation and be prominently related to response inhibition and sensory-motor integration. Importantly, beta activities have been associated with top-down processing regarding the maintenance of the current sensorimotor or cognitive state^26^ and the regulation of motor performance during tasks^69,70^. Frontal NoGo ERS activation at the beta band (see Fig. 1), which was suggested be involved in inhibitory motor control^65^ and stimulus-driven attention^71^, and the update of the adaptive response^72^, confirmed the malfunctions of frontal cognitive and inhibitory processing in PD. Furthermore, the UPDRS score was correlated with NoGo ERS power and onset latency, indicating that the reduction in amplitude and the delay in onset of ERS were associated with defective motor executive functions. This also suggests that altered NoGo ERS responses could characterise the deregulation of top-down sensorimotor integration and cognitive processing in PD.

As a preliminary investigation of Go/NoGo-related oscillatory alterations in PD, this study has several limitations. First, we used a 50%/50% ratio of Go and NoGo trials to ensure a sufficient number of NoGo trials to capture changes in power associated with the NoGo conditions. This was because the amplitude of beta ERD increased with the frequency of appearance of trial conditions^73^. However, there may be no enough Go trials to detect the differences between the PD patients and controls^74,75^. Second, the three-second interval between the warning cue and Go/NoGo stimuli might be not enough to prevent interferences between the neurophysiological responses induced by Go/NoGo stimuli and by the warning cue^28,74^. Third, owing to heterogeneity in symptoms and severity in PD, future studies should focus on larger patient cohorts with symptom- and severity-specific subgroups and explore neuropsychological measures such as the Wisconsin Card Sorting Test (WCST), which could yield data on the neural correlates of cognitive performance. Last, cognitive functions in PD patients might be influenced by their anti-parkinsonism medications, although cortical alterations have been reported even in drug-naive PD patients^76,77^.

## Conclusions

Patients with PD were characterised by delayed or attenuated ERD and ERS activities during the Go and NoGo conditions. These neurophysiological alterations might stem from abnormalities in motor programming, response inhibition, and frontal inhibitory modulation in PD. In particular, Go ERS was related to motor performance, while NoGo ERS was associated with motor symptoms, which could emerge from disrupted sensorimotor-cognitive interactions. Further extensive investigations are necessary to confirm the longitudinal treatment responses in PD.

## Materials and Methods

### Participants

Patients with PD were enrolled from both the Neurological Institute of Taipei Hospital and Taipei Veterans General Hospital. The diagnosis of PD was based on the UK Parkinson’s Disease Society Brain Bank clinical diagnostic criteria^78^. The clinical motor symptoms were scored with the Unified Parkinson’s Disease Rating Scale (UPDRS)^79^, while cognitive status was evaluated with the Mini-Mental State Examination (MMSE)^80^ at the day performing experiment. HC and patients with PD were all right-handed as assessed by a modified Edinburgh handedness inventory^81^, presented with normal physical and neurologic examinations, and were free of any history of systemic diseases or major neurological disorders. Twelve patients with PD (mean age: 65.75 ± 7.99 y/o; 9 males) and 13 age-matched healthy control subjects (mean age: 64.46 ± 4.24 y/o; 7 males) participated in this study. Before the experiment, all PD patients were on their usual anti-parkinsonism medications, such as an anticholinergic agent or monoamine oxidase inhibitor, but L-dopa and dopamine agonists were withdrawn for a period of at least 12 hours. Written informed consent was obtained from each participant, and this study was approved by the ethics committees of Taipei Hospital and Taipei Veterans General Hospital. All methods have been performed in accordance with the ethical standards laid down in the Declaration of Helsinki.

### Go/NoGo task

**Figure 5:**
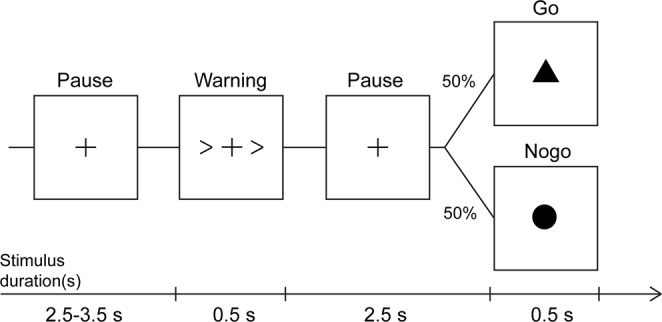
The experimental procedure of the Go/NoGo paradigm in this study.

The Go/NoGo task consisted of a warning visual cue (a pair of arrows) with a duration of 500 ms, a pause period (cross symbol) for 2500 ms, and symbols for Go (the triangle symbol) or NoGo condition (the circle symbol) for 500 ms (Fig. 5). The visual clue and symbols were presented at the centre of the screen within 2–4 degrees of visual angle. The participants were seated and asked to perform a self-paced extension of the right index finger at a 45° angle in response to the triangle stimuli (Go condition) as soon as possible while keeping the left hand at rest during the task. The Go condition occurred on 50% of trials, with the NoGo stimuli occurring on the remaining 50% of trials. Before recording, the participants completed a training session of 10 trials. In the experimental session, they performed a sufficient number of trials until approximately 50 correct responses for the Go and NoGo conditions were carried out. The average trials to reach sufficient numbers were 72.61 ± 14.99 in healthy controls and 99.67 ± 38.42 in patients in the Go condition and 83.08 ± 26.43 in healthy controls and 110.50 ± 38.61 in patients in the NoGo condition. The correct responses were defined as extension of the right index finger within 500 ms after the triangle stimuli disappeared in the Go condition and successful withholding any movement within 500 ms after circle stimuli disappeared in the NoGo condition. A finger-lift optical response pad (Art. no. NM20999N, Elekta Neuromag, Helsinki, Finland) was placed beneath the right hand, and the subject’s index finger was placed on the groove of the pad at the starting position. A light beam generated by a light source embedded in one side of the groove would be sensed by a detector on the other side. During the MEG recording, the subject was asked to comfortably place the right index finger on the groove; thus, the light beam was not sensed by the detector. When the subject extended the index finger, the detection of light beam triggered an event signal (<1 ms delay between response and recorded event). The onset of the event signal was defined as the onset time (t = 0 ms) of finger extension. The reaction time was measured as the time elapsed between the onset of triangle stimuli (Go condition) and the onset of finger extension. Reaction time and incorrect responses were recorded for further statistical analysis.

### MEG recording and data analysis

The MEG data during the Go/NoGo task were obtained in a magnetically shielded room with a 306-channel whole-head MEG system (VectorviewTM, Elekta Neuromag, Helsinki, Finland) that consisted of 102 identical triple sensor elements. In order to precisely localise the cortical activities, (1) four coils to stand for the head position were placed on the subject’s scalp, and their positions in the head coordinate frame specified by the nasion and two pre-auricular points were measured with a 3-dimension digitizer using Cartesian coordinates; (2) approximately 50 additional scalp points were also digitized; (3) these landmarks and points of the head position allowed for further registration of the MEG and magnetic resonance imaging (MRI) coordinate systems. The continuous MEG data were acquired with the signal digitization rate set to 500 Hz and the recording passband set to 0.1 to 160 Hz. The continuous MEG raw data were epoched from 3 s before the Go/NoGo stimuli to 3 s after the stimuli. Responses coincident with prominent vertical electro-oculogram signals (>300 µV) or MEG artefacts (>3000 fT/cm) were automatically rejected. Artefacts from the electrocardiogram were removed from the data using signal-space projection (SSP)^82^. Because our PD patients were akinetic-rigid dominant, the parkinsonian tremor was not often. When occasional parkinsonian tremor was presented during MEG recording, we stopped the recording by visual inspection and re-started the MEG recording after the tremor diminished. Moreover, epochs from an incorrect response to the Go/NoGo stimuli were also excluded from subsequent analyses.

The MEG data were decomposed using Morlet wavelet analysis implemented in the Brainstorm toolbox (http://neuroimage.usc.edu/brainstorm/), yielding a time-frequency map ranging from 1 to 60 Hz in frequency and −3 s to 3 s in time. ERD/ERS quantifies the oscillatory power change relative to a reference period of −3 to −2 s by the formula: ERD (or ERS) % = A-R/R × 100 (A = the power within the frequency band of interest during the active period of the event; R = mean power of the reference period)^83^. The single sensor with the maximal amplitude of beta ERD and ERS at the planar gradiometer directly over the sensorimotor cortex contralateral to the movement in the Go conditions was selected by visual inspection. The same sensor was also selected for the NoGo conditions. The onset latency of ERD and ERS is respectively defined as the first of 5 consecutive, significant values less and larger than the mean power during the reference period^84^.

Furthermore, the dynamic oscillatory ERD/ERS activation was mapped onto the individual’s T1-weighted MRI using weighted minimum norm estimate (MNE) analysis^30,31^. Head magnetic resonance images were obtained after MEG recording from 2016 to 2018. T1-weighted images were acquired using a 3T Siemens Trio system (3D MPRAGE T1 sequence, TR = 2530 ms, TE = 3.03 ms, TI = 1100 ms, recording matrix = 256 × 256 pixels, field of view = 256 mm, and slice thickness = 1 mm for the 192 slices). The forward problem utilised an overlapping spheres model that fits one local sphere for each sensor^85^ and then derives the density of a set of electric dipoles located at the cortical surface. The forward head-model was calculated from the MRI-derived surface model of each participant’s brain to describe the signal pattern generated by a unit dipole at each allowed location on the surface. The surface model was reconstructed from the T1-weighted structural volumetric images (BrainVISA 4.0.2, http://brainvisa.info).

### Statistical analysis

We used a chi-squared test to test the difference in categorical variables, such as gender. The Kolmogorov-Smirnov test was used to evaluate the data distribution. The data were normally distributed except for MMSE, peak frequency of the NoGo task, error rate in the NoGo task, and amplitude of the Go ERD. An independent t-test or a Mann-Whitney U test was performed to examine the difference between the PD patients and HC with respect to behavioural (reaction time, error rate, MMSE) and MEG (onset latency, peak frequency, and power change) responses. An independent t-test, which is a parametric test, was used to examine the data in a normal distribution. The Mann-Whitney U test, a nonparametric test, was used to examine the data in a non-normal distribution. Correlations between MEG response measures and behavioural data or clinical scores were estimated using Pearson’s correlation method. All numerical data were presented as the mean ± standard deviation (SD), unless specified otherwise. A p-value < 0.05 was considered statistically significant.

## Supplementary information

The results of Kolmogorov Smirnov tests and tests used for comparisons

Supplementary Dataset 1
