# Supplementary material for: Attenuated NoGo-related beta desynchronisation and synchronisation in Parkinson’s disease revealed by magnetoencephalographic recording
Source: Sci Rep. 2019 May 10;9:7235. doi: 10.1038/s41598-019-43762-x (PMC6510752; doi:10.1038/s41598-019-43762-x)
Supplement: Supplementary file 1 — The results of Kolmogorov Smirnov tests and tests used for comparisons [file 41598_2019_43762_MOESM1_ESM.pdf]

Attenuated NoGo-related beta desynchronisation and synchronisation in Parkinson's disease revealed by magnetoencephalographic recording

Hung-Ming Wu,[1,5,+] Fu-Jung Hsiao,[1,2,+] Rou-Shayn Chen,[6] Din-E Shan,[4] Wan-Yu Hsu,[1,7,8] Ming-Chang Chiang[9], Yung-Yang Lin[1,2,3,4,*]

[1]Institute of Brain Science, National Yang-Ming University, Taipei, Taiwan

[2]Brain Research Center, National Yang-Ming University, Taipei, Taiwan

[3]School of Medicine, National Yang-Ming University, Taipei, Taiwan

[4]Department of Neurology, Neurological Institute, Taipei Veterans General Hospital, Taipei, Taiwan

[5]Department of Neurology, Taipei Hospital, Ministry of Health and Welfare, Taiwan

[6]Department of Neurology, LinKou Chang Gung Memorial Hospital, Taoyuan, Taiwan

[7]Department of Neurology, [8]Neuroscape, University of California San Francisco, San Francisco, California, United States of America.

[9]Department of Biomedical Engineering, National Yang-Ming University, Taipei, Taiwan

[*]Corresponding author at: Department of Critical Care Medicine and the Neurological Institute, Taipei Veterans General Hospital, 201, Shihpai Rd Sec 2, Taipei, 112 Taiwan (ROC)

E-mail address: yylin@vghtpe.gov.tw

+Hung-Ming Wu and Fu-Jung Hsiao contributed equally to this article

	Kolmogorov Smirnov tests	Distribution/tests
Go reaction time	p>0.05	normal/ Independent t-test
MMSE	p<0.001	non-normal/ Mann-Whitney U test
Age	p>0.05	normal/ Independent t-test
Go error rate	p>0.05	normal/ Independent t-test
NoGo error rate	p=0.005	non-normal/ Mann-Whitney U test
peak frequency of Go task	p>0.05	normal/ Independent t-test
peak frequency of NoGo task	p<0.001	non-normal/ Mann-Whitney U test
Power of Go ERD	p=0.026	non-normal/ Mann-Whitney U test
Power of Go ERS	p>0.05	normal/ Independent t-test
Power of NoGo ERD	p>0.05	normal/ Independent t-test
Power of NoGo ERS	p>0.05	normal/ Independent t-test
Onset latency of Go ERD	p>0.05	normal/ Independent t-test
Onset latency of Go ERS	p>0.05	normal/ Independent t-test
Onset latency of NoGo ERD	p>0.05	normal/ Independent t-test
Onset latency of NoGo ERS	p>0.05	normal/ Independent t-test

Table 1: The results of Kolmogorov Smirnov tests and tests used for comparisons.
Note: ERD: event-related desynchronisation. ERS: event-related synchronisation.
